# A Strontium-Modified Titanium Surface Produced by a New Method and Its Biocompatibility *In Vitro*

**DOI:** 10.1371/journal.pone.0140669

**Published:** 2015-11-03

**Authors:** Chundong Liu, Yanli Zhang, Lichao Wang, Xinhua Zhang, Qiuyue Chen, Buling Wu

**Affiliations:** 1 Department of Stomatology, Nanfang Hospital, Southern Medical University, Guangzhou, P. R. China; 2 College of Stomatology, Southern Medical University, Guangzhou, P. R. China; 3 Department of Stomatology, the Second Affiliated Hospital of Guangzhou Medical University, Guangzhou, P. R. China; Université de Lyon—Université Jean Monnet, FRANCE

## Abstract

**Objective:**

To present a new and effective method of producing titanium surfaces modified with strontium and to investigate the surface characteristics and *in vitro* biocompatibility of titanium (Ti) surfaces modified with strontium (Sr) for bone implant applications.

**Materials and Methods:**

Sr-modified Ti surfaces were produced by sequential treatments with NaOH, strontium acetate, heat and water. The surface characteristics and the concentration of the Sr ions released from the samples were examined. Cell adhesion, morphology and growth were investigated using osteoblasts isolated from the calvaria of neonatal Sprague-Dawley rats. Expression of osteogenesis-related genes and proteins was examined to assess the effect of the Sr-modified Ti surfaces on osteoblasts.

**Results:**

The modified titanium surface had a mesh structure with significantly greater porosity, and approximately5.37±0.35at.% of Sr was incorporated into the surface. The hydrophilicity was enhanced by the incorporation of Sr ions and water treatment. The average amounts of Sr released from the Sr-modified plates subjected to water treatment were slight higher than the plates without water treatment. Sr promoted cellular adhesion, spreading and growth compared with untreated Ti surfaces. The Sr-modified Ti plates also promoted expression of osteogenesis-related genes,and expression of OPN and COL-І by osteoblasts. Ti plates heat treated at 700°C showed increased bioactivity in comparison with those treated at 600°C. Water treatment upregulated the expression of osteogenesis-related genes.

**Conclusions:**

These results show that Sr-modification of Ti surfaces may improve bioactivity *in vitro*. Water treatment has enhanced the response of osteoblasts. The Sr-modified Ti heat-treated at 700°C exhibited better bioactivity compared with that heated at 600°C.

## Introduction

Titanium and its alloys have been widely used in medical applications such as dental and orthopedic implants because of their excellent mechanical properties, osseointegration properties and superior biocompatibility[1, 2].The success rates of dental implants are highly dependent on the quality of the bone-implant interface as well as factors such as patient age, sex, bone quality and site of implantation. Osteoporosis is a common bone metabolic disorder characterized by microarchitectural deterioration and low bone mass [3], which affects the aging population especially postmenopausal women. Although clinical studies show that osteoporosis is not a contraindication for the use of dental implants, a longer healing period is required to achieve osseointegration[4–7], and animal experiments show that poor bone density has an inhibitory effect[8–11].

Many drugs have been administered to increase the success rates of dental implants in osteoporotic bone and to enhance osteogenesis after implantation[9, 10, 12, 13].Strontium ranelate(SR) has been successfully used in osteoporotic patients as an anti-osteoporosis drug[14–17], and studies show that oral administration of SR can improve implant osseointegration and enhance early implant fixation in osteoporotic animals[18, 19] and normal rats[20].

Strontium(Sr) promotes bone formation, and reduces osteoclast activity and bone resorption[21–24].Long-term local and targeted delivery of an optimal dose Sr to the implant tissue interface is considered a good approach to increase implant osseointegration and avoid the potential adverse effects associated with oral administration[25, 26]. Sr has been incorporated into coatings on implants as strontium-doped hydroxyapatite(HA)[27]. Sr-incorporated Ti oxide surfaces obtained by hydrothermal methods significantly enhanced osteoblast function[28] and promoted biomechanical fixation of implants in a rabbit model[29].

The deposition of strontium-doped HA, anodic oxidation and hydrothermal treatments all require special apparatus and are not suitable for devices with a complicated structure. Alkaline-heat treatment can create a bioactive surface on Ti and Ti alloy implants and appears clinically promising[30].Titanium and its alloys treated sequentially with sodium hydroxide(NaOH) and calcium chloride(CaCl_2_) exhibit enhanced apatite-forming ability *in vitro* and superior biomechanical performance *in vivo*[31–33].

Sr is located in the same group in the periodic table of elements as calcium(Ca). Although Sr and Ca have similar chemical, physical, and biological characteristics, unlike Ca, Sr is able to inhibit bone resorption[34].In this study we developed a biomimetic coating using simple chemical treatment with NaOH, Sr acetate and heat-treatment in order to modify the titanium surface topography and chemistry to render the surface biocompatible. We demonstrate that Sr-modified Ti surfaces considerably enhance adhesion, spreading and growth of bone cells. The microstructure and composition of their surfaces were characterized, and the cytocompatibility of the Sr-modified surfaces was also investigated using osteoblasts isolated from neonatal Sprague-Dawley rats.

## Materials and Methods

### 2.1 Fabrication of Sr-modified Ti plates

Titanium plates 10mm×10mm×1mm were fabricated from commercially pure grade 2 titanium (purity >99.85%)(Queen Titanium Co., Baoji, China). Ti plates were mirror polished using grit SiC abrasive paper (grain sizes from #400 to #1200) and then ultrasonically cleaned for 30 min with acetone, absolute ethyl alcohol, and deionized water sequentially to remove contaminants related to manufacturing and handling.

The plates were soaked in 5M sodium hydroxide solution in sealed plastic bottles at 60°C for 24h. After soaking, they were gently rinsed with deionized water for 30s and dried for 1 h at 60°C in an electric oven. The alkaline-treated samples were subsequently immersed in 0.1M strontium acetate solution at 40°C for 24h, then washed and dried as described before. Next, they were heated to 600°C (Sr600) or 700°C(Sr700) at a rate of 5°C/min and maintained at temperature for 1h, followed by natural cooling in a muffle furnace in air. After heat treatment, half the samples were soaked in deionized water at 80°C for 24h, then washed and dried. Untreated plates were used as controls (Table 1).

**Table 1:** Treatment groups for the titanium plates.

Group	Treatments
Ti	untreated (control group)
Sr600	NaOH+strontium acetate +600°C
Sr600W	NaOH+ strontium acetate +600°C+water
Sr700	NaOH+ strontium acetate +700°C
Sr700W	NaOH+ strontium acetate +700°C+water

### 2.2 Surface characterization

The surface of the samples was observed by field emission scanning electron microscopy (FE-SEM) (S-4300, Hitachi Co, Tokyo, Japan) at an accelerating voltage of 10KV,with an energy-dispersive X-ray (EDX) spectrometer (Genesis 60S, EDAX Company, Mahwah, NJ, USA). The hydrophilicity of the titanium surface was measured by the contact angle of 1μL H_2_O using a contact angle measuring device (OCA 20LHT, Dataphysics, Stuttgart, Germany).Three samples were used for each measurement.

### 2.3 Sr ions release

The Sr-modified Ti samples were soaked in 2mL of phosphate-buffered saline(PBS) in a sealed bottle at 37°Cfor1 day, taken out, and then immersed again in 2mLof fresh PBS. This process was repeated for a total of 7 days. The released Sr concentrations in the PBS were measured by inductively-coupled plasma mass spectrometry (ICP-MS, Agilent Technologies, Santa Clara, CA, USA). Three samples were used in this experiment and the mean value was used for analysis.

### 2.4 Cell cultures, cell morphology, cell adhesion and growth

Ti surfaces were tested *in vitro* using calvarial osteoblasts from neonatal (2–3 days old) Sprague-Dawley rats (Laboratory animal center of Southern Medical University, Guangzhou, China), isolated by trypsin and collagenase digestion. This experiment was performed in accordance with the Guidelines provided by the Animal Care and Use Committee of Southern Medical University. The protocols were approved by the Animal Care and Use Committee of Southern Medical University(Guangzhou, China).The rats were killed by cervical dislocation and the osteoblasts were isolated as previously described[35].The cells were cultured in Dulbecco’s modified Eagle’s medium (Hyclone, Logan, UT, USA)with low glucose containing 10% fetal bovine serum (Hyclone) at 37°C in a carbon dioxide incubator with medium changes every 2–3 days. Cells of passage 2–4 were used in the experiments.

The samples were placed in 24 well plates and the osteoblasts were seeded at a density of 8×10^4^/well for the cell adhesion assay and 4×10^4^/well for the other assays unless otherwise mentioned. Plates were sterilized by 60Co gamma-irradiation at a dose of 25kGy. Six samples were analyzed for cell morphology, cell adhesion and growth.

Cell morphology was studied by fluorescence imaging of the cells on different plates. The cells were inoculated at a density of 2×10^4^/well for 24 h and then fixed for 15 min in 4% (w/v) paraformaldehyde. After fixation, they were washed in PBS, permeabilized in 3% (v/v) Triton X-100 in deionized water for 5 min, washed again and then stained with TRITC-Phalloidin (YEASEN, Shanghai, China) to visualise the actin cytoskeleton, then counterstained with DAPI (Sigma, St. Louis, MO, USA) to visualize cell nuclei. The images were captured using an inverted fluorescence microscope.

For cell adhesion assays, the cells were seeded onto plates and cultured for 1, 2 or4 hours, then the plates were removed, gently washed with PBS to remove non-adherent cells, then placed into new 24-well plates for assessment of cell numbers using the Cell Counting Kit-8 assay (CCK-8, Dojindo Molecular Technologies, Japan) in accordance with the manufacturer’s instructions. For growth assays, the cells were cultured on plates for 1, 2, 3, 5 and 7 days and cell numbers were assessed using the CCK-8assay.

### 2.5 Expression of osteogenesis-related genes and proteins

The expression levels of osteogenesis related genes were measured using qRT-PCR. The cells were seeded on plates at a density of 4×10^4^cells/well, cultured for 7 daysor14 days, then harvested using TRIzol(Life Technologies, Carlsbad, CA, USA) to extract the RNA. The RNA was reverse transcribed into complementary DNA (cDNA) using PrimeScript RT reagent Kit (Takara, Kusatsu, Japan) and qRT-PCR analysis was performed on a Roche Light Cycler 480using SYBR Premix Ex Taq II(Takara). The primers for the target genes are listed in Table 2. The expression levels of the target genes were normalized to that of the housekeeping gene β-actin.

**Table 2:** The target genes and primer used for Quantitative Real-time PCR.

Gene	Primer sequences
β-actin	F5’-TGAAGTACCCCATTGAACACGG
β-actin	R5’-GGGTCATCTTTTCACGGTTGG
collagen type I	F5’-GGAGAGAGCATGACCGATGG
collagen type I	R5’-AAGTTCCGGTGTGACTCGTG
Runx2	F5’-AGCGGACGAGGCAAGAGTTT
Runx2	R5’-GGGTTCTGAGGCGGGACA
OPN	F5’-AGCATTCTCGAGGAAGCCAG
OPN	R5’-AGTGTTTGCTGTAATGCGCC

The expression of osteopontin (OPN) and collagen type- І (COL- І)on different surfaces was examined by western blotting after 7 days of culture. Osteoblasts were rinsed with cold PBS then harvested by lysis in radio-immunoprecipitation assay (RIPA) buffer with protease inhibitors. Total protein was determined using the MicroBCA protein assay kit (Cowin Biotech, Beijing, China). Blots were probed with antibodies to OPN (1:800,BS-0019R,Bioss, Shanghai, China) and Collagen-І(1:800, ab90395, Abcam, Cambridge, MA, USA)with anti-actin (1:1,000, sc-1616r, Santa Cruz Biotechnology, Santa Cruz, CA, USA) as the internal control. Cells were harvested and frozen at -80°C before use. Three samples were used in qRT-PCR and western blotting.

### 2.6 Statistical analysis

All data are expressed as mean ± standard deviation and are investigated by using the Shapiro-Wilk normality test. Comparisons between groups were analyzed by Kruskal-Wallis test (when the data are non-normal distribution) and one-way analysis of variance (ANOVA),least significant difference (LSD)post hoc test (when the variance was regular)and Dunnett T3 test post hoc test(when the variance was irregular). All differences were considered significant when *P* < 0.05.

## Results

### 3.1 Surface characterization

**Fig 1:**
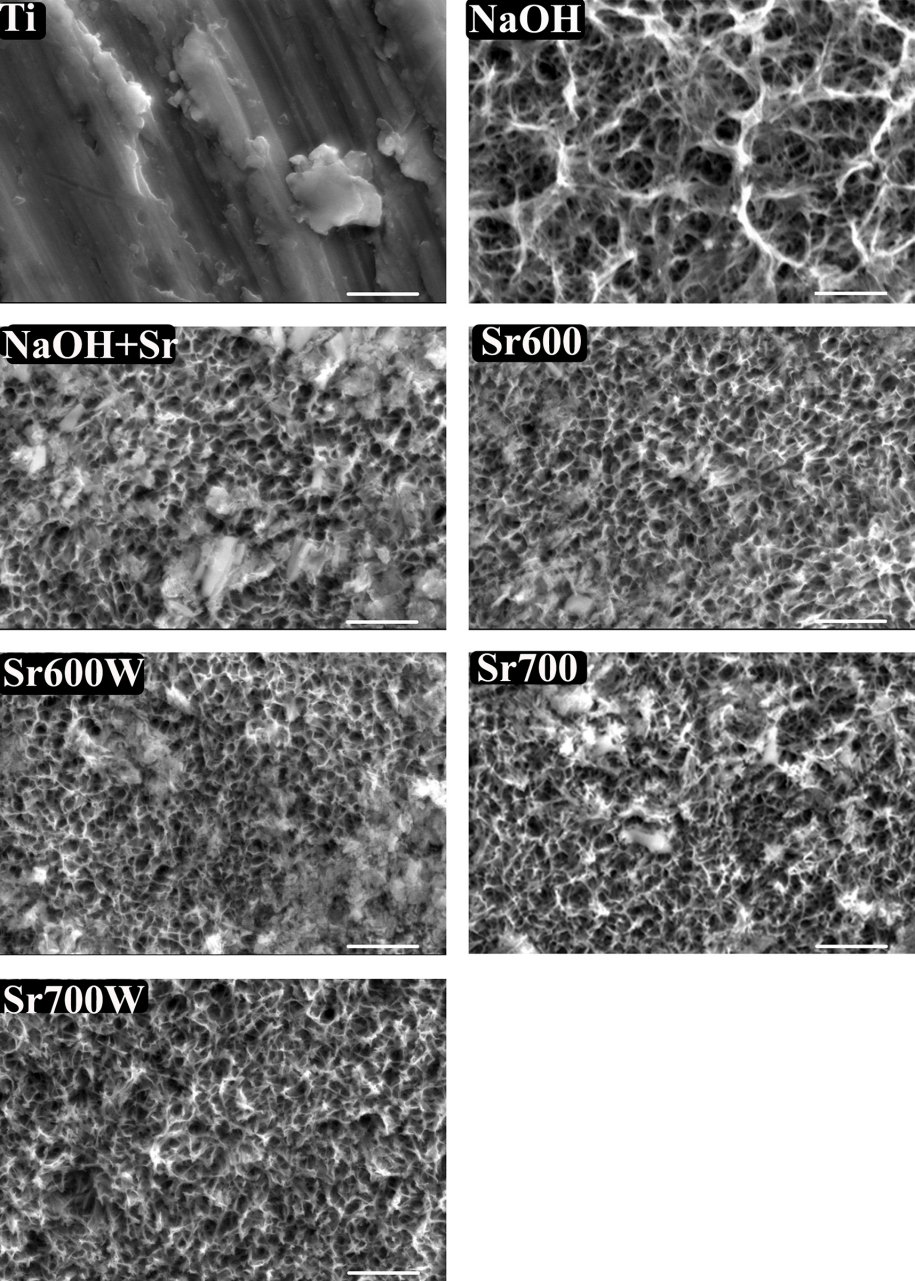
FE-SEM images of the surfaces of Ti plates subjected to different treatments (20000×, bar = 1μm).

FE-SEM photographs of the surfaces of the Ti plates shows uniform porous structure at the nanometer scale after NaOH treatment. There was a notable difference in surface morphology after Sr acetate treatment, with smaller pore size, thicker and more distinct walls and some deposits, this appearance was essentially unchanged by the subsequent heat and water treatments (Fig 1).

The EDX results (Table 3) showed thatapproximately8.48 ± 0.13at.% of Na was incorporated into the surface of the Ti after NaOH treatment. The Na ions were completely replaced with Sr and approximately 5.37 ± 0.35 at.% of Sr was incorporated into the surface of the Ti by subsequent Sr treatment. The amount of Sr incorporated was almost unchanged by heat treatment but decreased slightly after water treatment.

**Table 3:** Surface atomic concentrations of Ti samples subjected to sequential treatment with NaOH, Sr acetate, heat, and water, analyzed by EDX (at.%, $\bar{\text{x}}\pm \text{s}$, n = 3).

Treatment	Ti	O	Na	Sr	C	Al
Ti	91.78±0.92	3.60±0.09	0	0	3.21±0.93	1.42±0.08
NaOH	33.09±1.09	43.23±0.47	8.48±0.13	0	13.82±1.47	0.64±0.13
NaOH+Sr	30.16±2.55	46.65±1.44	0	5.37±0.35	16.19±1.68	1.63±1.07
Sr600	34.69±1.48	46.18±1.74	0	4.82±0.72	13.02±1.06	1.29±0.11
Sr600W	34.09±0.85	46.51±1.64	0	4.83±0.89	12.83±1.50	0.94±0.21
Sr700	33.28±0.56	46.75±0.66	0	5.63±0.10	13.87±1.10	0.47±0.09
Sr700W	35,50±1.23	47.62±0.45	0	4.19±0.39	12.12±0.78	0.58±0.21

### 3.2 Hydrophilicity and roughness of the surfaces

**Fig 2:**
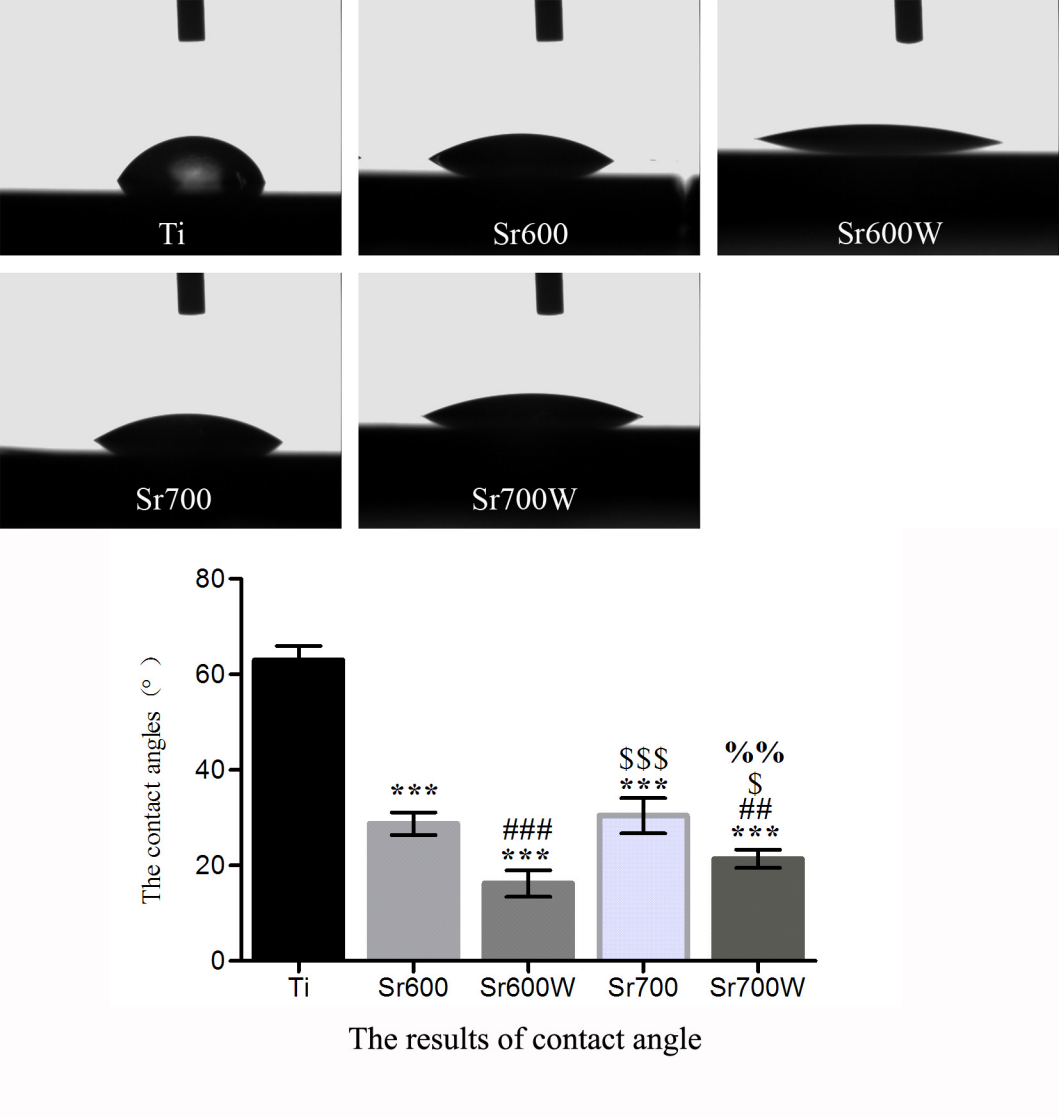
Images of H_2_O droplets pipetted onto the plates and the results of contact angle. The contact angles of the Sr-modified plates were lower than Ti plates, and the contact angles were reduced by water treatment. (***P < 0.001 vs Ti; ##*P*< 0.01, ###*P*< 0.001 vs Sr600; $ *P*< 0.05, $ $ $*P*< 0.01 vs Sr600W; %% *P*< 0.01 vs Sr700).

The contact angles of a water droplet on the Ti and the Sr-modified Ti plate surfaces (Fig 2, Table 4) were62.9°, 28.7°, 16.2°, 30.4°and 21.4°respectively, indicating that the hydrophilicity of the Ti plates was increased by the incorporation of Sr ions. In addition the surface hydrophilicity was improved further by water treatment.

**Table 4:** The contact angle (°) of different surfaces (n = 3).

Group	contact angle
Ti	62.933±3.001
Sr600	28.733±2.359*
Sr600W	16.200±2.773* #
Sr700	30.400±3.727* $
Sr700W	21.400±1.900* # %

*P< 0.05 vs Ti

# *P*< 0.05vs Sr600

$ *P*< 0.05 vs Sr600W

%*P*< 0.05 vs Sr700).

### 3.3 Sr ion release

**Fig 3:**
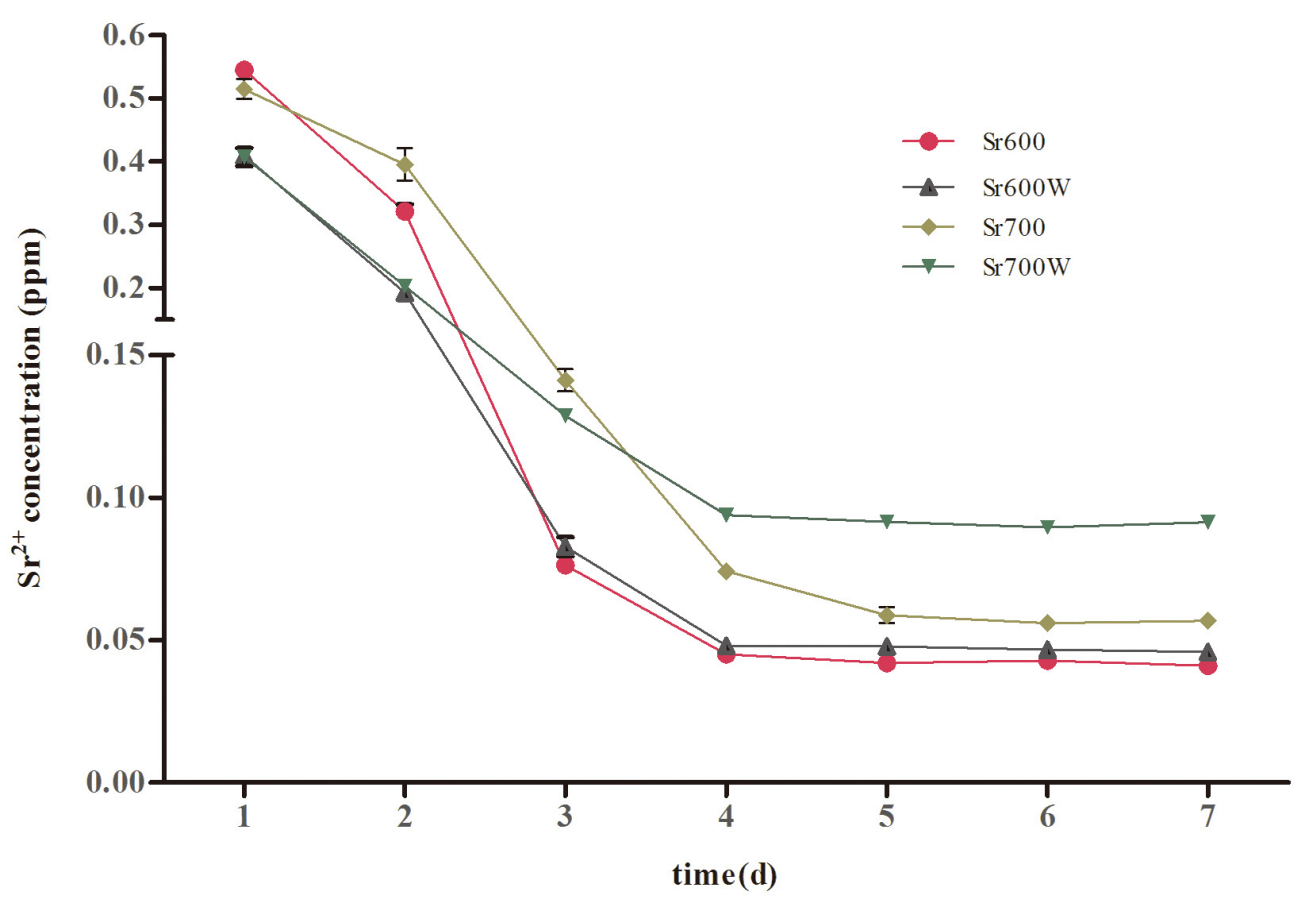
Concentrations of Sr ions measured by ICP-MS, which was released from the Sr-modified plates between day one and day seven.

Fig 3 shows the concentration of the Sr ions released from the Sr-modified plates into PBS. It can be seen in Fig 3 that Sr600 and Sr700 released 0.545 ppm and 0.514 ppm of Sr ions within 1 d respectively, then rapidly declined up to 4 days later. In contrast, the Sr600W and Sr700W group released 0.409ppm and 0.407ppm of Sr ions within 1 d respectively, then the released Sr amounts remained relatively constant after 3 days. The average Sr amounts released from Sr700 and Sr700W group are slight higher than Sr600 and Sr600W group with the exception of the burst release at day 1 and day 2. Over all the average amounts released from the Sr-modified plates subjected to water treatment were slight higher than the plates without water treatment.

### 3.4 Osteoblast morphology and adhesion

**Fig 4:**
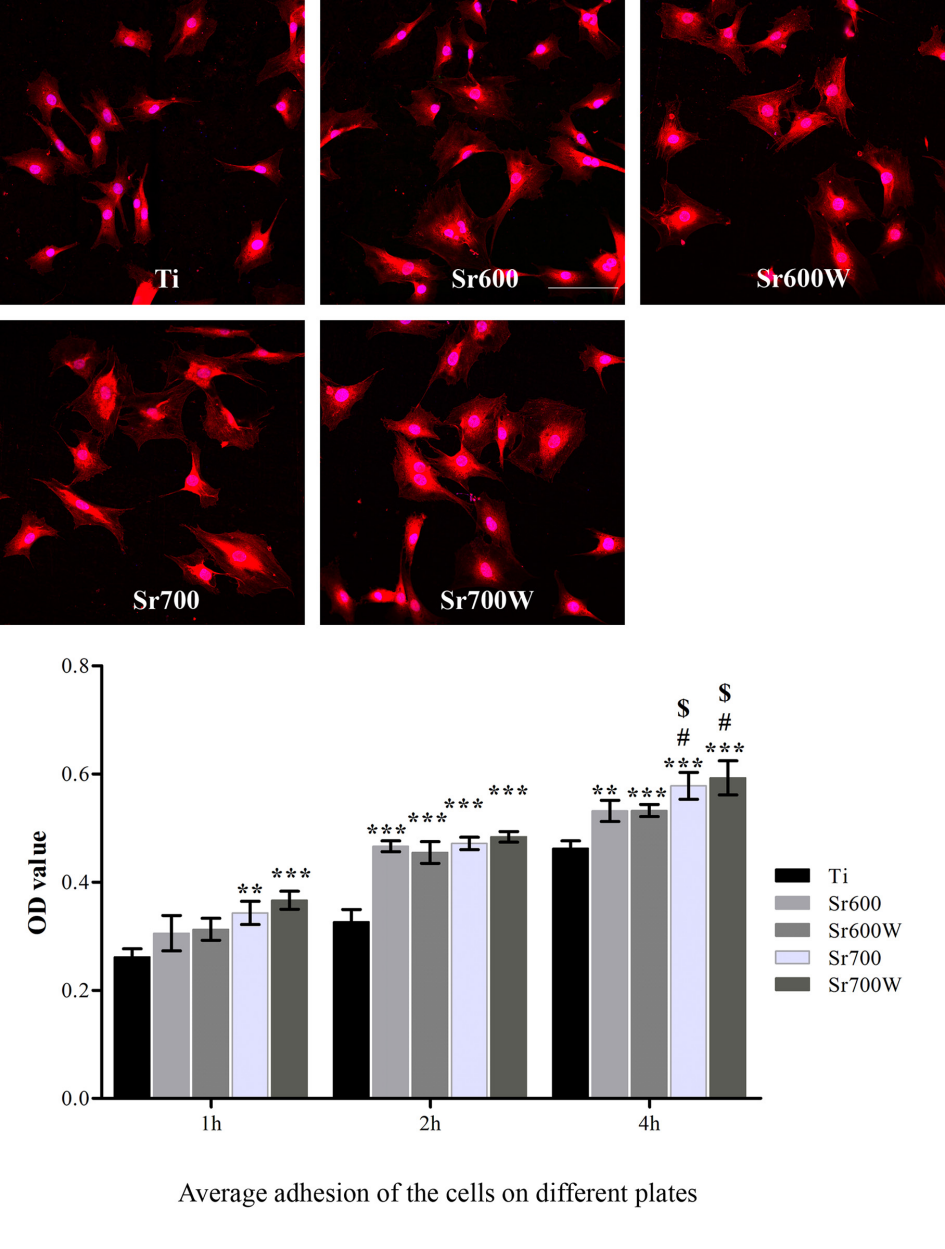
Fluorescence images of osteoblasts after 24 h of culture (×200) and the average adhesion of the cell on different platesafter 1, 2, or 4 h of culture. (*P< 0.05, **P< 0.01, ***P< 0.001 vs Ti; # *P*< 0.05, ## *P*< 0.01 vs Sr600; $ *P*< 0.05, $ $ *P*< 0.01 vs Sr600W).

**Fig 5:**
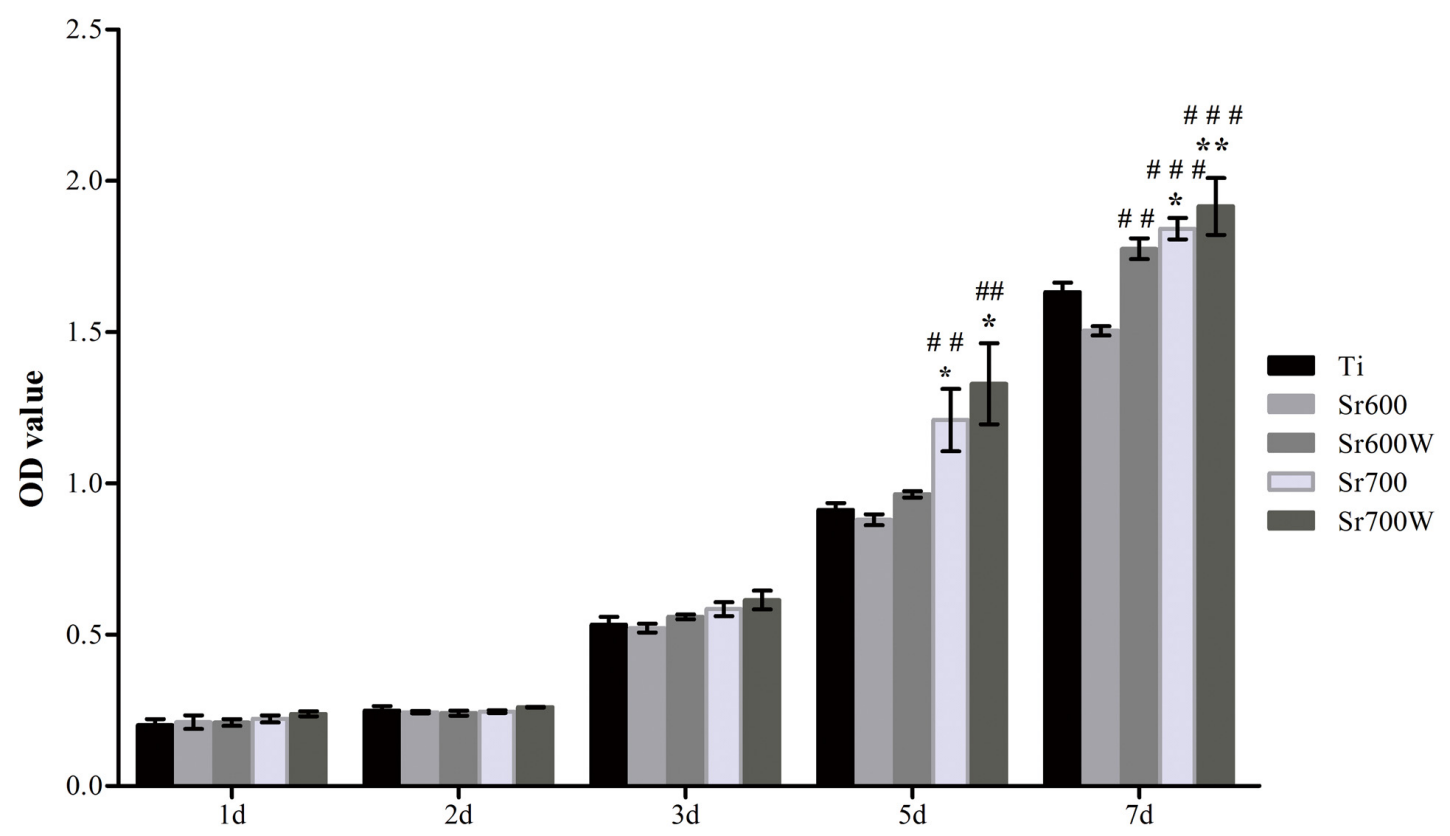
Cell growth on the different surfaces at 1, 2, 3, 5, and 7 days analyzed by CCK-8 assay. (*P< 0.05 Ti, **P < 0.01 vs Ti; # *P*< 0.05, ##*P*< 0.01, ###*P*< 0.001 vs Sr600; $*P*< 0.05, $ $*P*< 0.01, $ $ $*P*< 0.001 vs Sr600W).

Cell morphology is closely related to the function of osteoblasts. After 24h, differences in cell shape could be observed(Fig 4). The cells on the Sr-modified surfaces were more extended than those on Ti surfaces, while there was no significant difference in cell spreading among the Sr-modified surfaces. Initial cell adhesion is known to be the key step controlling cell proliferation and differentiation on biomaterials[36]. Adhesion of osteoblasts to the different surfaces was determined after 1, 2 and 4 h (Fig 5). The number of adherent cells increased with time on all five surfaces. Cell adhesion was significantly higher on the Sr-modified surfaces than on Ti at all time-points. There was no obvious difference in the initial adherent cell number on plates before and after water treatment. The number of adherent cells on the Sr700W surfaces was significantly higher than on the Sr600 and Sr600W surfaces at 1 h and 4 h.

### 3.5 Growth of osteoblasts

The number of cells was determined on the different plates up to 7 days of culture (Fig 5). No significant difference was observed between surfaces at 1, 2 and 3 days. The number of cells increased with time on all five surfaces but growth was more rapid on the Sr-modified surfaces than on Ti surfaces especially on the Sr700W group. These results indicated that Sr incorporation into the Ti surface improved osteoblast growth.

### 3.6 Expression of osteogenesis-related genes and proteins

**Fig 6:**
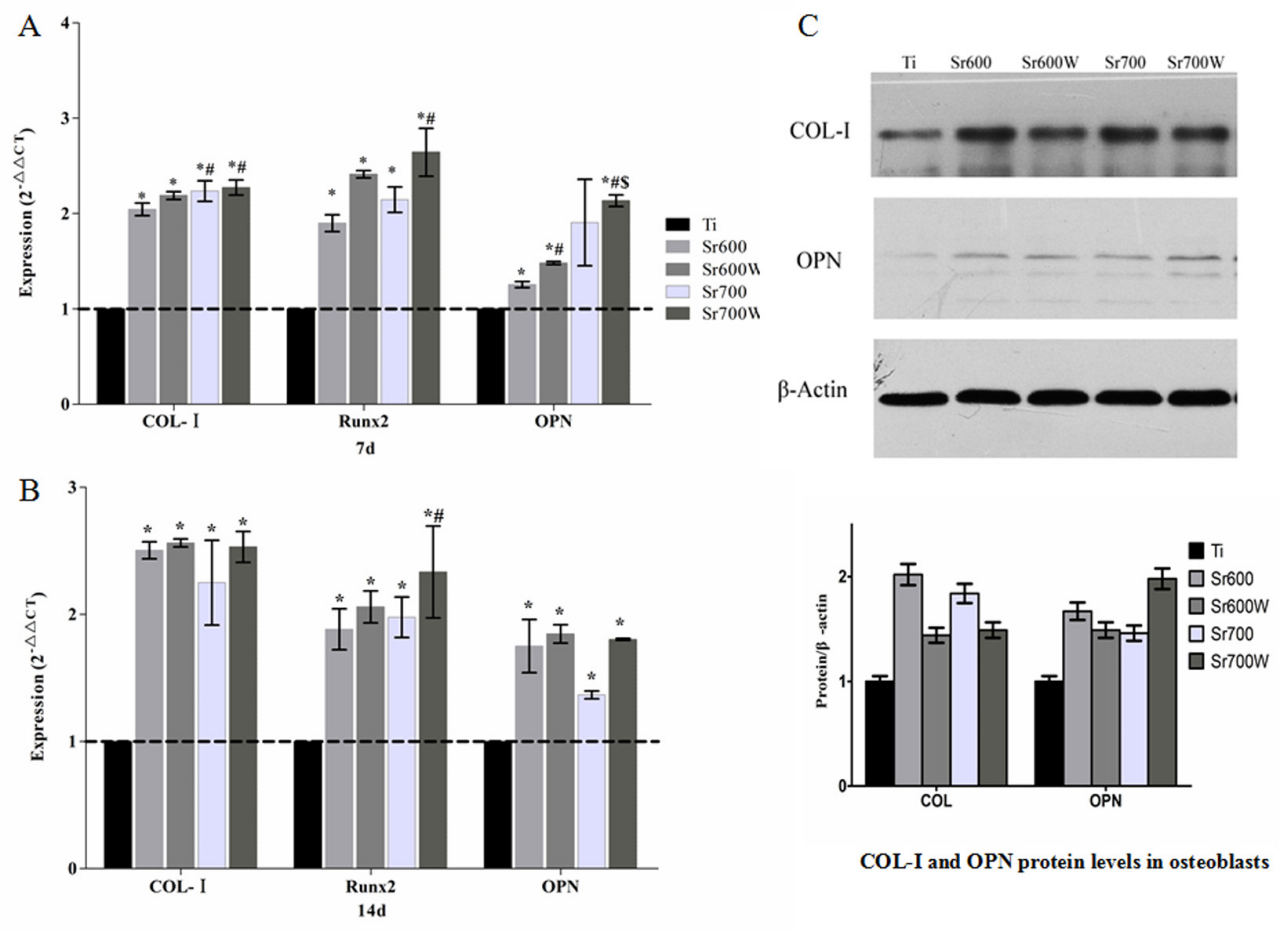
Expression of osteogenesis-related genes and proteins. (A) Expression of Col-1, Runx2and OPN by osteoblasts cultured on different substrates for 7days.(B) Expression of Col-1, Runx2and OPN by osteoblasts cultured on different substrates for 14 days.(C) COL-I and OPN protein levels in osteoblasts grown on different surfaces for 7 days, analyzed by western blot. Beta-actin served as loading control. Sr incorporation increased expression of both COL-I and OPN. (*P< 0.05 vs Ti; # *P*< 0.05 vs Sr600; $ *P*< 0.05 vs Sr600W; %*P*< 0.05vs Sr700).

Analysis of the expression of osteogenesis-related genes showed that the Sr-modified plates significantly increased the expression of osteogenesis-related genes including Col-І, Runx2 and OPN (Fig 6A and 6B). Further, expression of Runx2 and OPN was higher in cells grown on Sr600Wsurfaces than on Sr600 surfaces at day 7. The Sr700W surfaces showed higher OPN expression compared with the Sr700 surfaces (Fig 6A). This suggests that water treatment results in a significant enhancement of the expression of osteogenesis-related genes.

It has been suggested that matrix osteogenic factors are closely associated with bone remodeling *in vitro*. COL-І and OPN might therefore play important roles in bone mineralization and remodeling. Using western blotting, we found that the levels of COL-І and OPN were upregulated by NaOH, strontium acetate, heat, and water treatments (Fig 6C). And OPN was more highly expressed in the Sr700W group compared with the other groups.

## Discussion

In this study, Sr ions were incorporated into Ti surfaces by simple method. In previous studies, researchers have incorporated Sr ions into the surfaces of implants using various methods[37, 38]. However, the techniques mentioned in these studies require expensive specialized apparatus or a specialized high pressure apparatus and the results will also vary along with different parameters. In this study a simple method involving chemical, heat and water treatments using aqueous solutions was used to treat the surface of implants with regular and irregular structure. This method is controllable and does not need any special apparatus. Furthermore, in present study, *in vitro* increased bioactivity of Sr-modified surfaces was confirmed.

We investigated the surface characteristics and biocompatibility of Sr-modified Ti surfaces using osteoblasts. When NaOH-treated Ti was subsequently soaked in a solution of strontium acetate, the Na ions were all replaced with Sr ions and a fairly large number of strontium ions were easily incorporated into the Ti surface(Table 3). This is due to formation of a sodium hydrogen titanate layer on the Ti plates after NaOH treatment[39], the Na ions in which are easily replaced by Sr ions. The amount of Sr increased very little even if a solution of a higher concentration was used, since all of the Na was replaced by Sr in the 0.1 M Sr acetate solution. Quantification by EDX can give indications but not absolute values. In addition, most carbon is present as surface contaminants[40].These treatments changed the morphology of the surface as well as changing its chemical composition and crystalline structure. All these results show that this simple method effectively incorporates Sr ions into Ti surfaces. The present results show that a uniform porous structure is formed at the nanometer scale after treatment, and this structure enhances *in vitro* cytocompatibility as shown by cell morphology, adhesion and growth assays.

In this study, the Sr-modified plates were soaked in 2mL of PBS to determine Sr release and only 1mL cell culture medium was used in cell study, so the ultimate concentrations of released Sr ions acting on the osteoblasts was above the lower limit of the Sr concentration in another study[41], in which 0.21–21 μg/mL strontium ranelate (0.071–7.1 ppm of Sr) added to culture medium significantly promoted differentiation of human bone marrow mesenchymal stem cells *in vitro*. Park *et al*. found that Sr-modified Ti6Al4V fabricated by hydrothermal treatment, which released Sr ions at concentrations of 0.103–0.135 ppm enhanced ALP activity and gene expression without improving cell attachment and proliferation[29].The substrate surface properties, such as roughness, chemical composition and wettability, influence cell behavior[42].The hydrophilicity of the Ti plates was promoted by the incorporation of Sr ions and water treatment. It is reasonable to suggest that surfaces properties and the released Sr ions may contribute to the increased bioactivity in this study *in vitro*.

Sr-modified Ti surfaces improved the spreading of osteoblasts, increased the early adherent cell number and enhanced osteoblast growth, and the results of Sr700 and Sr700W groups were better than those of the Sr600 and Sr600W groups. Zhao *et al*. and Panzavolta *et al*. found that Sr has a slight positive effect on the initial adherent cell number[37, 43]. In addition Sr incorporation into biomaterials has been previously observed to promote spreading of osteoblasts[38, 44]. Previous reports have shown dose-dependent effects of Sr on cell proliferation [45, 46]. Sr incorporation into biomaterials has previously been shown to promote spreading of osteoprecursor cells and osteoblasts[44, 47] and to enhance bone formation *in vitro* and *in vivo*[48, 49]. Incorporation of Sr ions into a Ti surface obtained by hydrothermal treatment promoted osteogenesis and reduced osteoclastogenesis *in vivo*[26]. However a Sr-containing Ti-6Al-4V surface produced by hydrothermal treatment did not promote cell attachment or proliferation of MG63 cells compared with untreated Ti–6Al–4V[29]. The different findings from different studies may be due to the different Sr amounts used and its release kinetics. We found that more Sr ions released from Sr700 and Sr700W group than from Sr600 and Sr600W group. And there were no obvious difference between surfaces topography before and after water treatment, possibly because the porous structure of the surfaces was essentially unchanged and the amount of Sr ions was changed little after water treatment.

In this study, Sr-modified Ti surfaces significantly increased expression of osteogenesis-related genes and proteins. These results are in accordance with previous *in vitro* studies on the effect of Sr incorporation into various materials[45]. Studies have shown that the effect of strontium incorporation on osteoblastic differentiation and mineralization does not increase with increasing Sr concentration[50].This suggests that when the concentration of Sr reaches a certain level there is no further stimulatory effect on osteoblastic cells. The expression of OPN and COL-І was also increased in Sr-modified groups compared with untreated Ti plates in this study. These results are in accordance with those of other studies reporting a stimulatory effect of Sr on osteoblastic cell behaviors [38, 41, 51][38, 41, 51]. Col-1 is an early marker of bone mineralization (ECM formation) while ALP and Runx2 are expressed later during mineralization phase. OPN is fully expressed in the late mineralization and remodeling phase[52]. The Sr-modified plates significantly promote the expression of osteogenesis-related genes including Col-І, Runx2 and OPN, demonstrating excellent bioactivity. Further experiments may be necessary to elucidate the detailed signaling pathway and mechanism involved. The present results indicate a dose dependent effects of Sr on osteoblasts and our results are consistent with previous reports[45, 46]. However it is plausible to suggest that differences in the release of Sr ions contribute to the improved bioactivity of the Sr-modified Ti plates *in vitro*.

Ti plates heat treated at 700°C enhanced the response of osteoblasts compared with those treated at 600°C. However, the FE-SEM and EDX results showed no significant difference. This may be explained by the fact that the average Sr amounts released from the Sr700 and Sr700W group are slight higher than the Sr600 and Sr600W group. In present study, water treatment did not significantly enhance adhesion, growth of the osteoblasts cultured on the surfaces, but significantly improved their gene expression. The water treatment may increase mobility of the Sr ions in the surfaces by a partial exchange of the Sr ions with the H_3_O^+^ions in the water, as in the case of the calcium titanate and magnesium-containing calcium titanate that formed on Ti[53, 54]. In addition the hydrophilicity increased significantly after water treatment and the Sr-modified Ti plates showed an increase in the release of Sr ions.

Based on these results, it is expected that Sr-modified Ti plates fabricated by this method would release Sr ions which would promote osteoblastic cell behaviors *in vitro*. Further detailed study will be needed to reach definite conclusions on these effects *in vitro and in vivo*.

## Conclusion

Sr-modified Ti plates can be obtained by simple treatment which is suitable for objects of arbitrary geometry. We characterized the physiochemical properties of Sr-modified Ti plates and evaluated cell attachment and cytocompatibility using osteoblasts. Water treatment significantly enhanced the expression of osteogenesis-related genes. Ti plates heat treated at 700°C showed better cytocompatibility and improved osteoblastic cell behaviors on their surfaces compared to those heated at 600°C. Although further detailed studies *in vitro* and *in vivo* are needed to elucidate the effects of these surfaces on bone responses, especially the Sr700 and Sr700W group, these results indicate that Sr-modified Ti has the potential for future use as a substrate for bone implants.

## Supporting Information

S1 Fig FE-SEM images of the surfaces of Ti plates subjected to different treatments. (ZIP)

S2 Fig Images of H2O droplets pipetted onto the plates and the results of contact angle. (ZIP)

S3 Fig Concentrations of Sr ions measured by ICP-MS. (ZIP)

S4 Fig Fluorescence images of osteoblasts after 24 h of culture and the average adhesion of the cell on different plates. (ZIP)

S5 Fig Cell growth on the different surfaces at 1, 2, 3, 5, and 7 days analyzed by CCK-8 assay. (ZIP)

S6 Fig Expression of osteogenesis-related genes and proteins. (ZIP)
