# Supplementary figures and images for: A Strontium-Modified Titanium Surface Produced by a New Method and Its Biocompatibility In Vitro
Source: PLoS One. 2015 Nov 3;10(11):e0140669. doi: 10.1371/journal.pone.0140669 (PMC4631518; doi:10.1371/journal.pone.0140669)

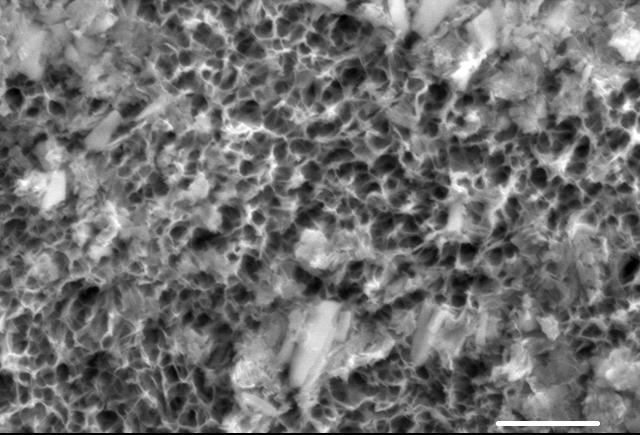

Supplement: S1 Fig — (ZIP) [file pone.0140669.s001.zip › S1 Fig 1 FE-SEM images of the surfaces of Ti plates subjected to different treatments/NaOH+Sr.tif]

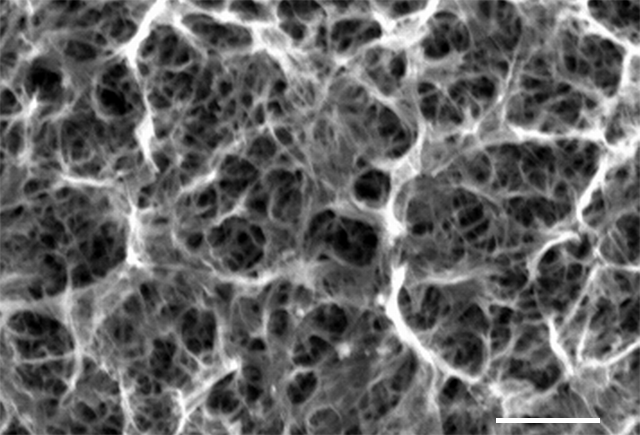

Supplement: S1 Fig — (ZIP) [file pone.0140669.s001.zip › S1 Fig 1 FE-SEM images of the surfaces of Ti plates subjected to different treatments/NaOH.tif]

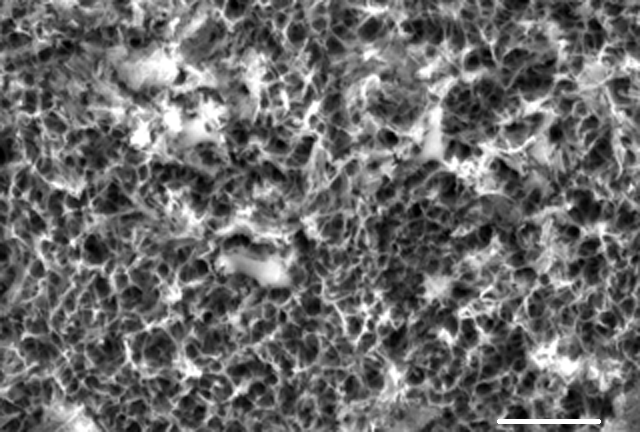

Supplement: S1 Fig — (ZIP) [file pone.0140669.s001.zip › S1 Fig 1 FE-SEM images of the surfaces of Ti plates subjected to different treatments/Sr 700.tif]

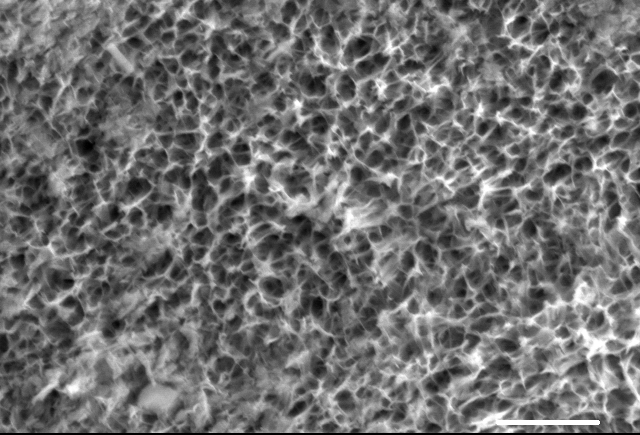

Supplement: S1 Fig — (ZIP) [file pone.0140669.s001.zip › S1 Fig 1 FE-SEM images of the surfaces of Ti plates subjected to different treatments/Sr600.tif]

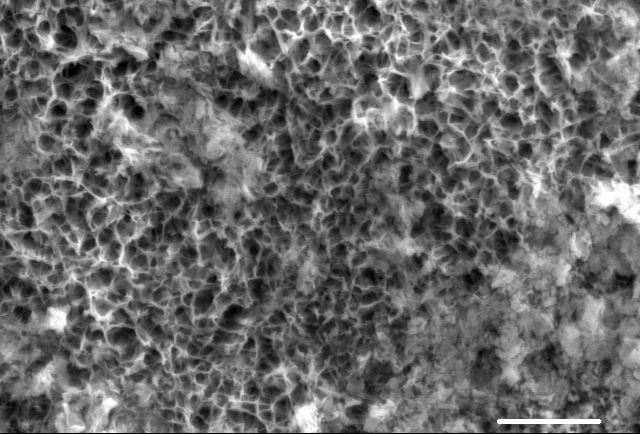

Supplement: S1 Fig — (ZIP) [file pone.0140669.s001.zip › S1 Fig 1 FE-SEM images of the surfaces of Ti plates subjected to different treatments/Sr600W.tif]

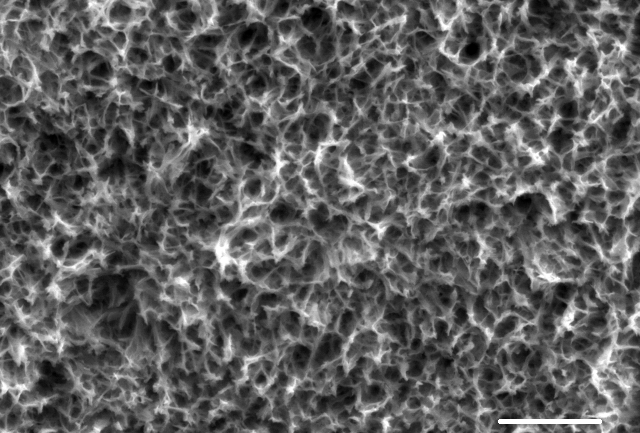

Supplement: S1 Fig — (ZIP) [file pone.0140669.s001.zip › S1 Fig 1 FE-SEM images of the surfaces of Ti plates subjected to different treatments/Sr700W.tif]

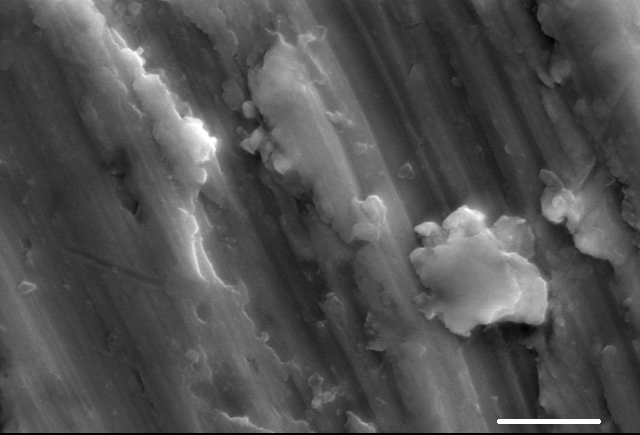

Supplement: S1 Fig — (ZIP) [file pone.0140669.s001.zip › S1 Fig 1 FE-SEM images of the surfaces of Ti plates subjected to different treatments/Ti.tif]

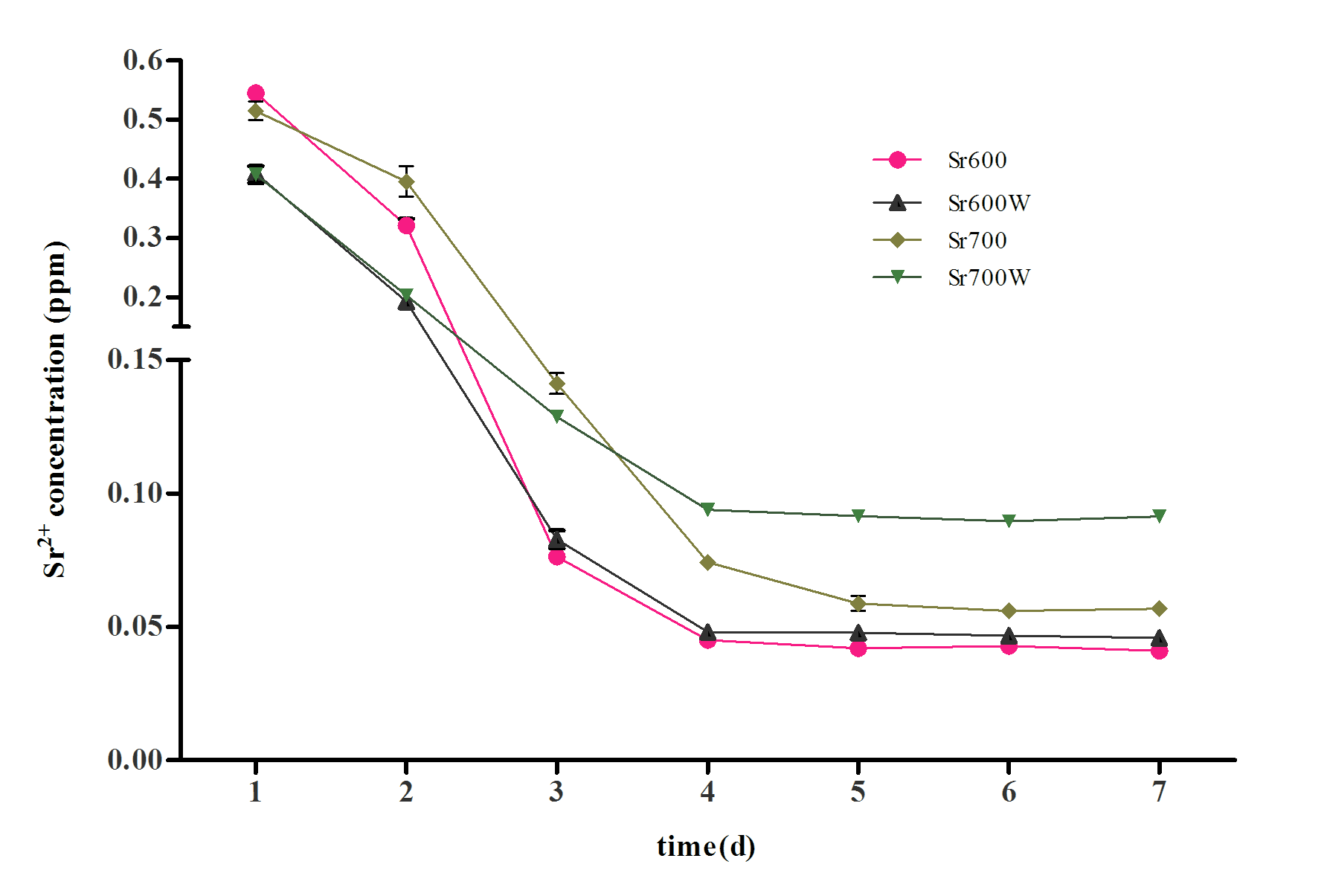

Supplement: S3 Fig — (ZIP) [file pone.0140669.s003.zip › S3 Fig 3 Concentrations of Sr ions measured by ICP-MS/Figure 3 Concentrations of Sr ions measured by ICP-MS.tif]

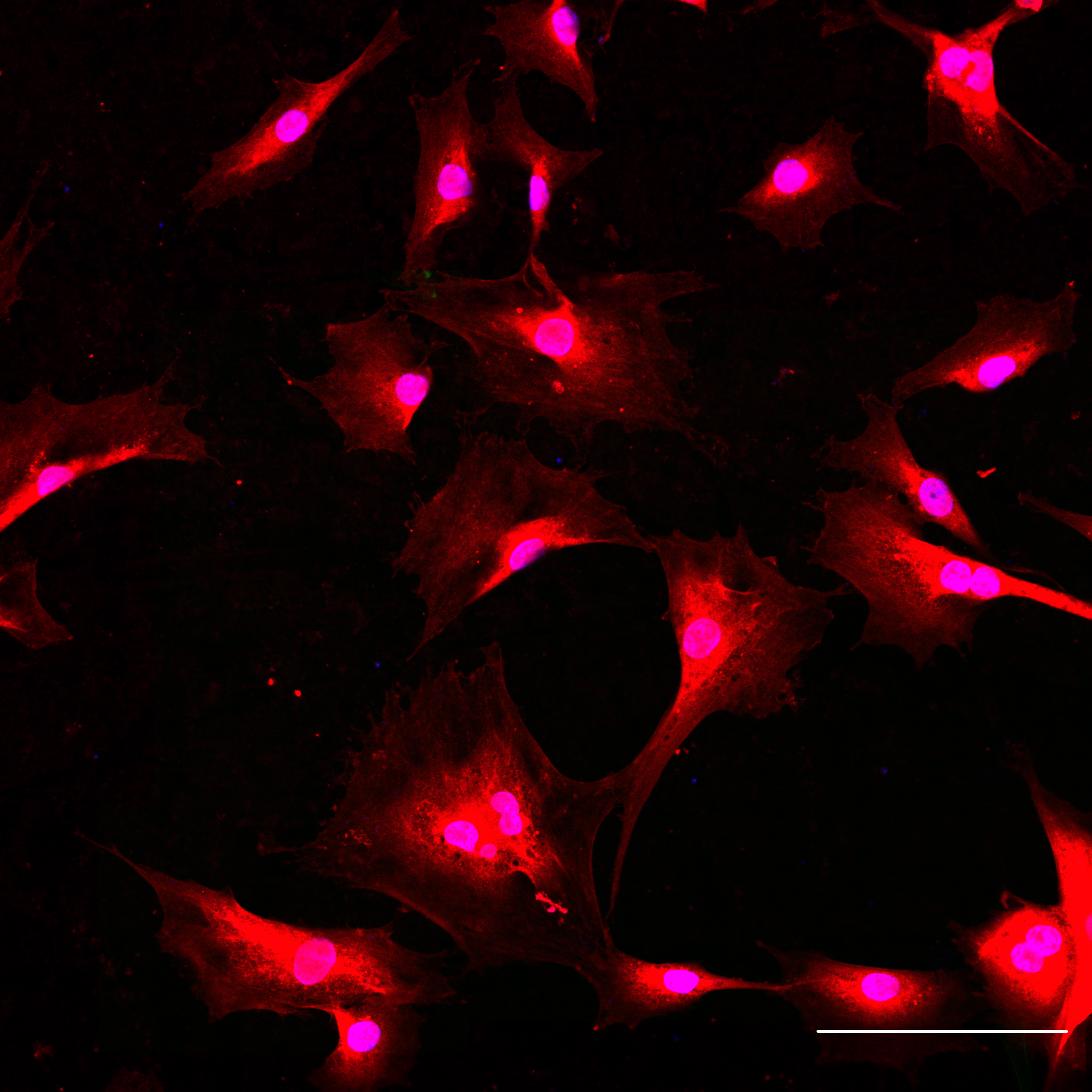

Supplement: S4 Fig — (ZIP) [file pone.0140669.s004.zip › S4 Fig 4 Fluorescence images of osteoblasts after 24 h of culture and the average adhesion of the cell on different plates/Sr600.tif]

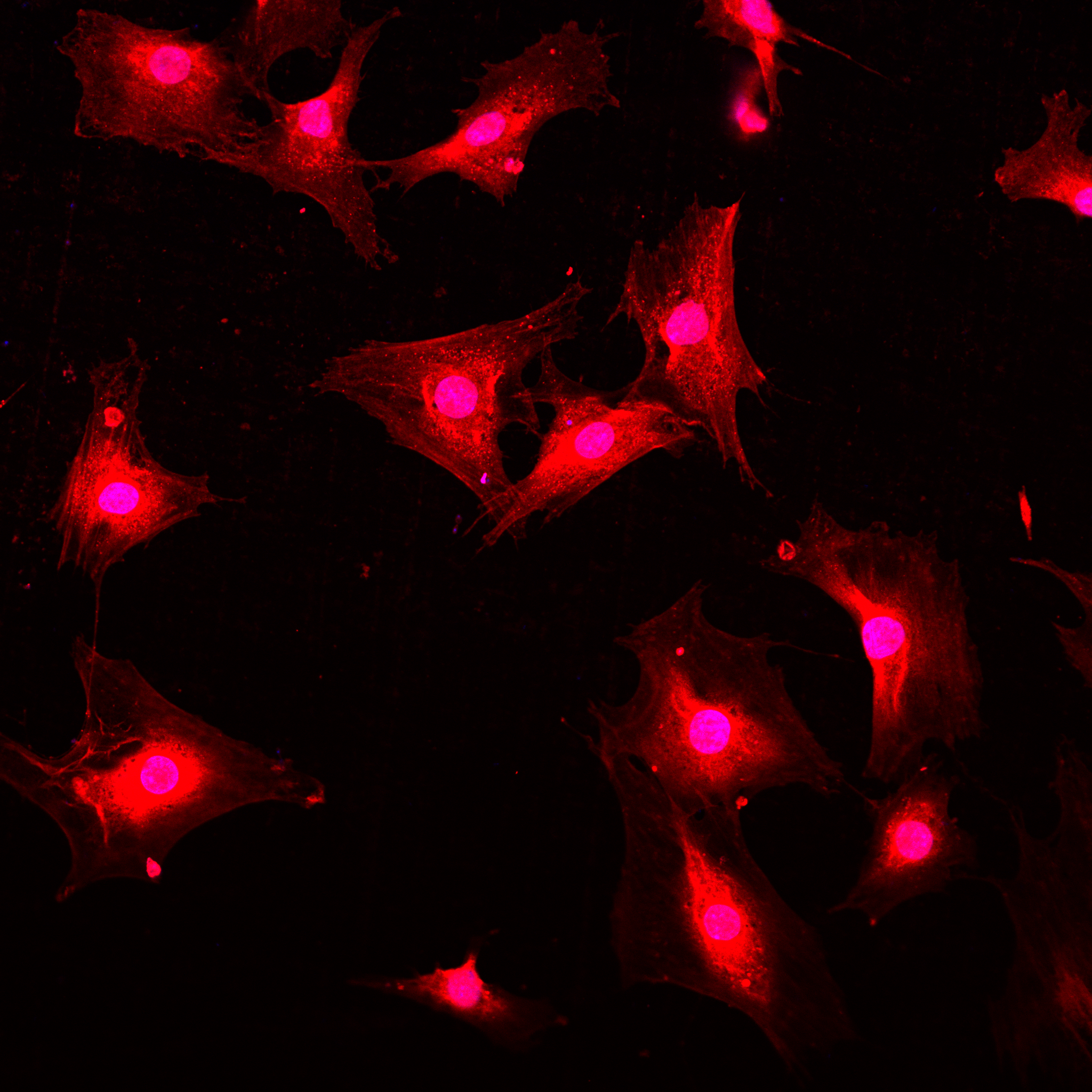

Supplement: S4 Fig — (ZIP) [file pone.0140669.s004.zip › S4 Fig 4 Fluorescence images of osteoblasts after 24 h of culture and the average adhesion of the cell on different plates/Sr600W.tif]

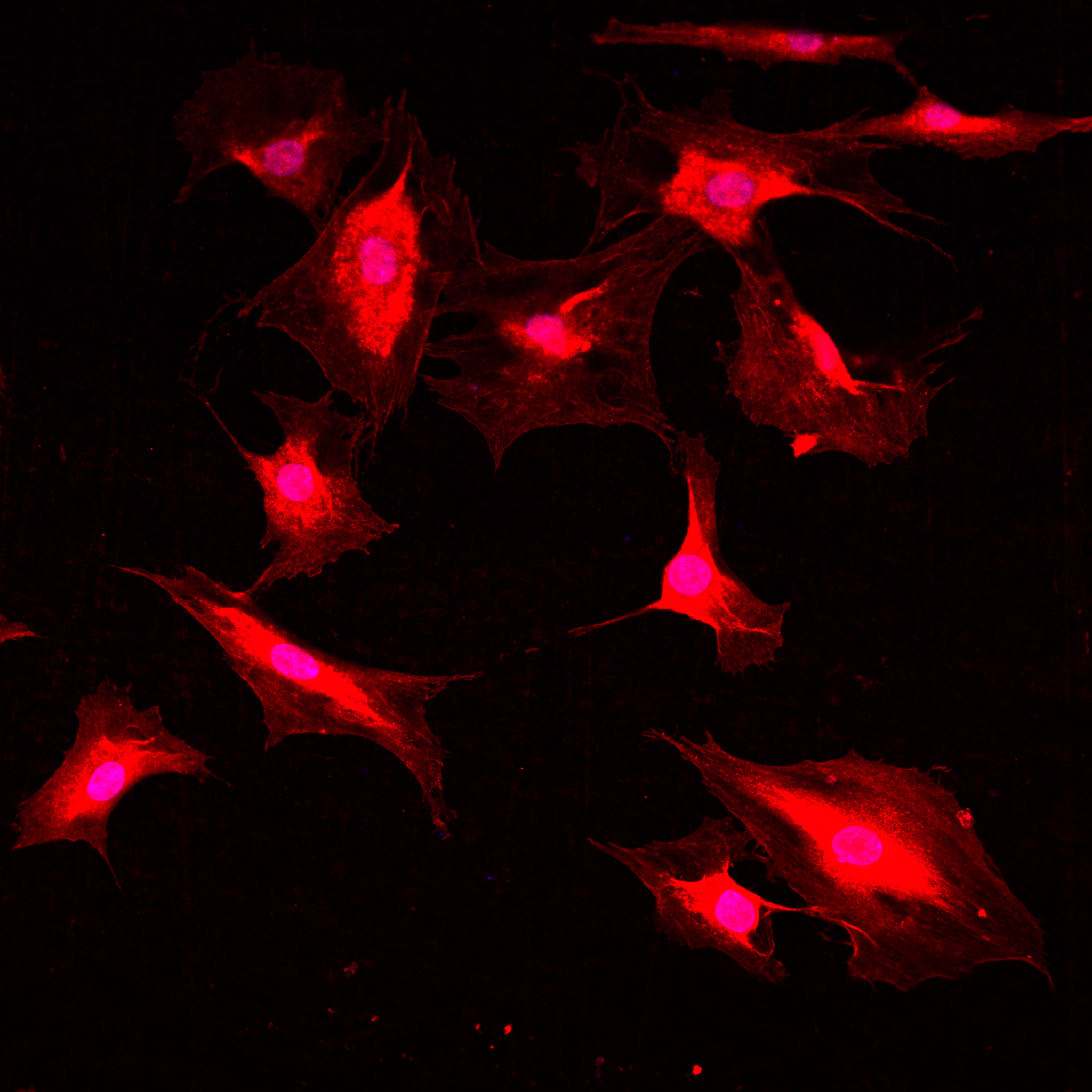

Supplement: S4 Fig — (ZIP) [file pone.0140669.s004.zip › S4 Fig 4 Fluorescence images of osteoblasts after 24 h of culture and the average adhesion of the cell on different plates/Sr700.tif]

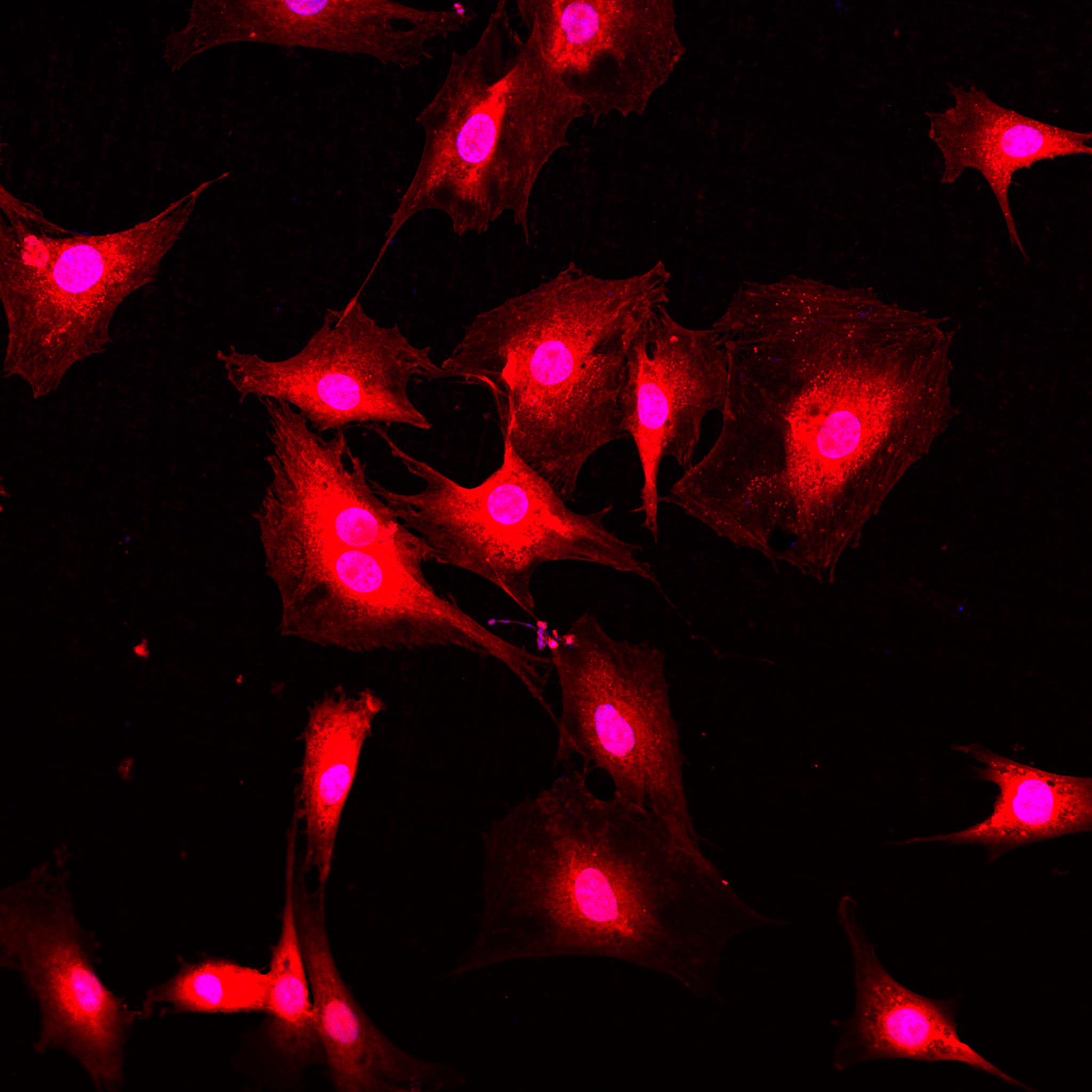

Supplement: S4 Fig — (ZIP) [file pone.0140669.s004.zip › S4 Fig 4 Fluorescence images of osteoblasts after 24 h of culture and the average adhesion of the cell on different plates/Sr700W.tif]

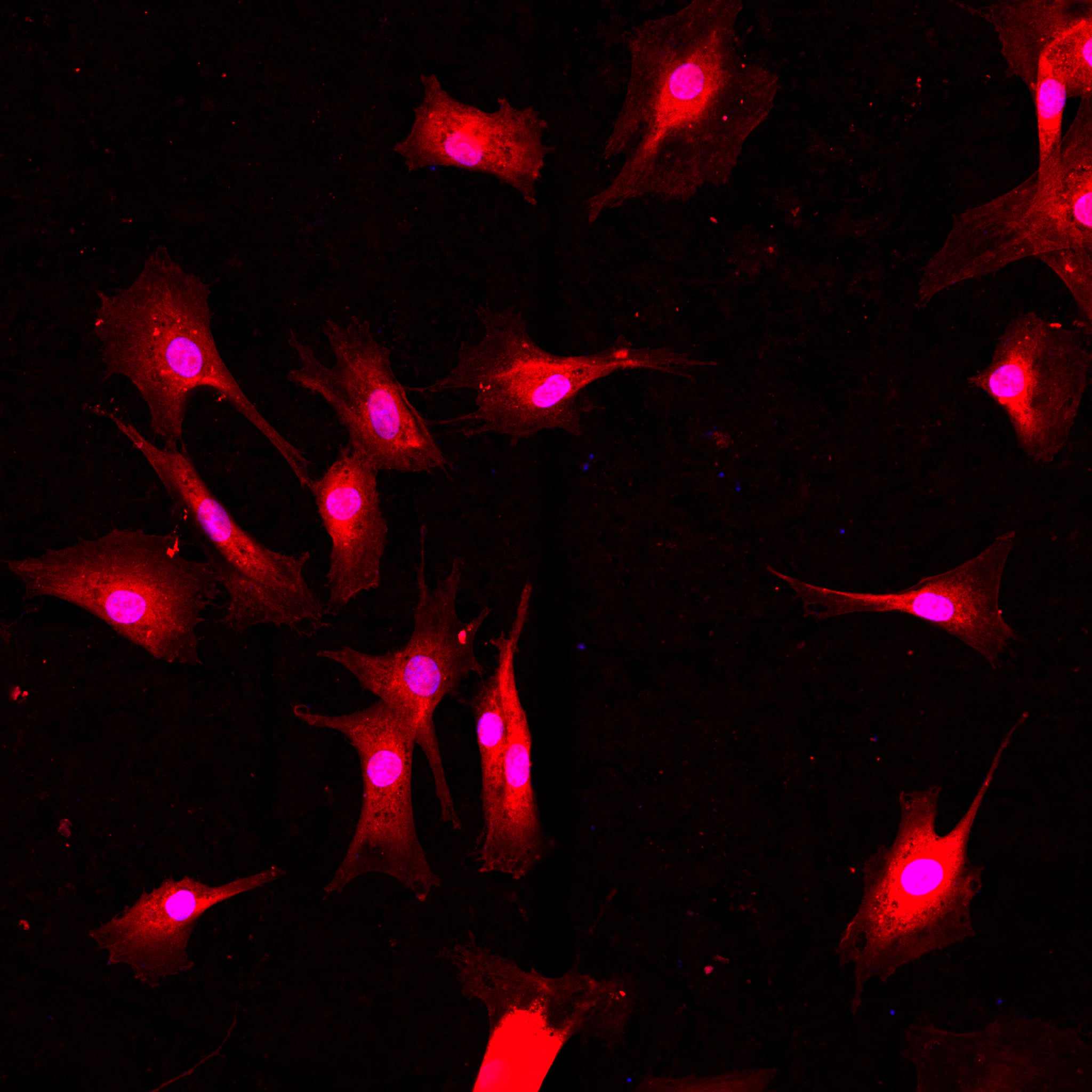

Supplement: S4 Fig — (ZIP) [file pone.0140669.s004.zip › S4 Fig 4 Fluorescence images of osteoblasts after 24 h of culture and the average adhesion of the cell on different plates/Ti.tif]

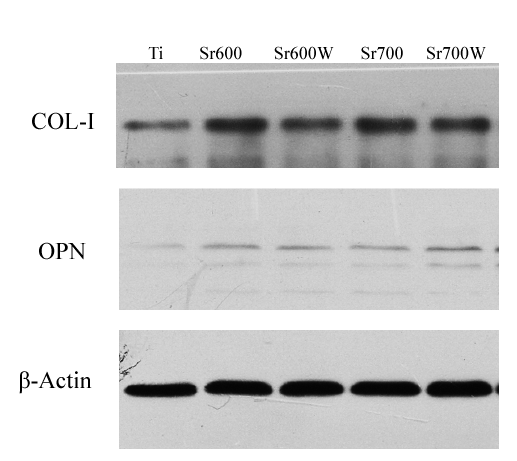

Supplement: S6 Fig — (ZIP) [file pone.0140669.s006.zip › S6 Fig 6 Expression of osteogenesis-related genes and proteins/WB.tif]
